# Editorial: Mechanistic theories of aging

**DOI:** 10.3389/fragi.2025.1617783

**Published:** 2025-05-12

**Authors:** John Tower

**Affiliations:** Molecular and Computational Biology Section, Department of Biological Sciences, University of Southern California, Los Angeles, CA, United States

**Keywords:** mitochondria, dietary restriction, gonad, mother’s curse, SAI, SKN-1, biomembranes, air pollution

## Introduction

**FIGURE 1:**
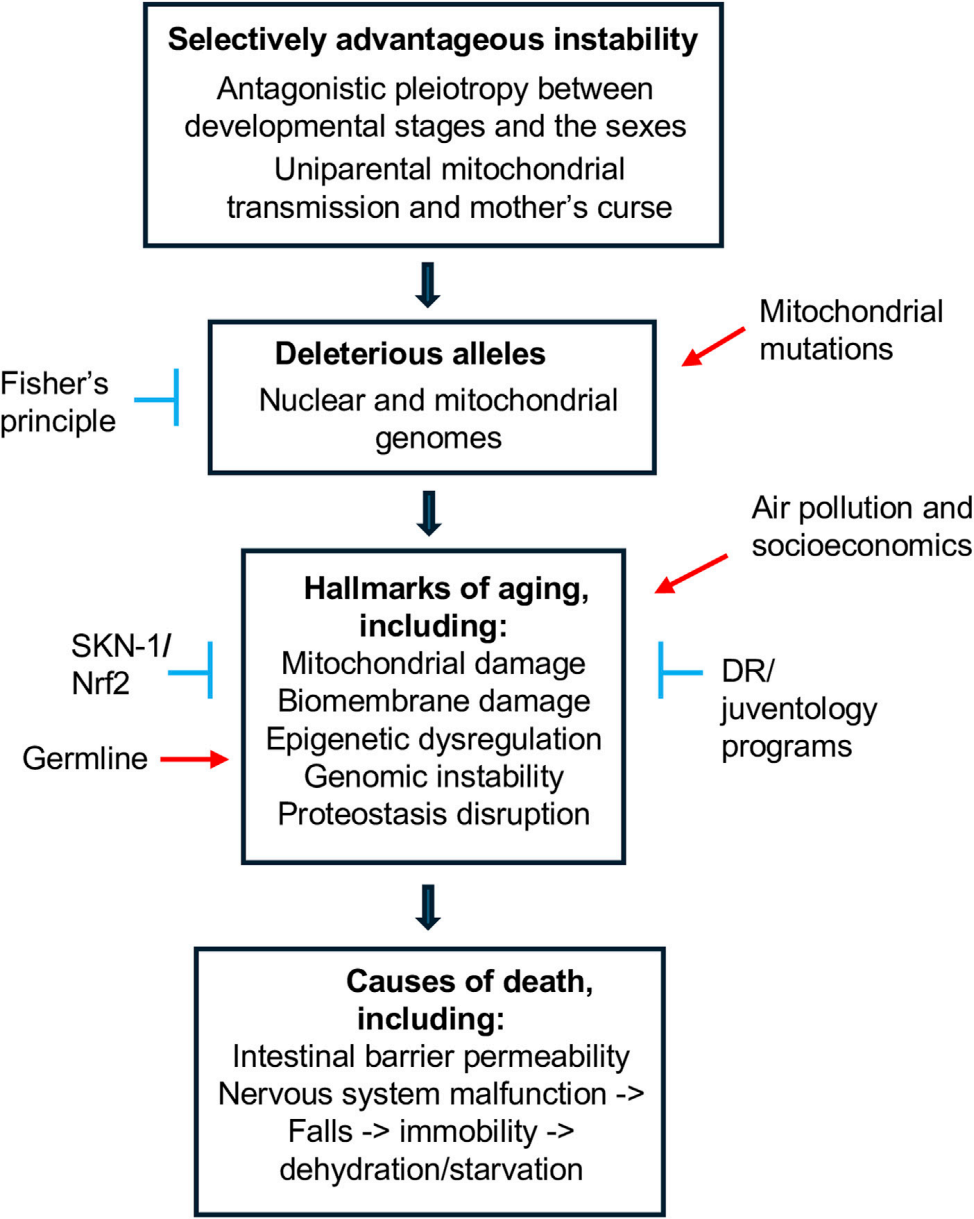
Outline of mechanistic theories of aging.

The genetic theory of aging involving mutation accumulation and antagonistic pleiotropy has relatively broad support, but does not specify mechanisms. Recent progress in the study of aging in humans and model systems has provided insight into mechanistic theories of aging. Because there is no universally accepted definition of aging, an operational definition of aging is key to attempts to define aging mechanisms. This Research Topic presents a series of articles that examine mechanistic theories of aging at every level of biological organization, ranging from molecules to genes, cells, organisms and society (Figure 1).

Mitochondrial dysfunction is one of 12 proposed “hallmarks” of aging, where aging is broadly defined as “…the time-dependent functional decline that affects most living organisms … ” (Lopez-Otin et al., 2013). The hallmarks are phenotypes that manifest with age and that can accelerate aging when experimentally increased, and in some cases, decelerate aging when targeted with therapeutic interventions. The hallmarks include mitochondrial dysfunction, genomic instability, loss of proteostasis, chronic inflammation, disabled macroautophagy, and others. These hallmarks may represent independent or semi-independent mechanisms that each contribute to aging. Alternatively, it might be argued that each hallmark results from a central mechanism involving mitochondrial maintenance failure.

## Main text

The review on selectively advantageous instability (SAI) (Tower) surveys the ways that molecular instability can provide a selective advantage to cells and organisms, above and beyond the generation of energy or mobilization of building blocks. Here, aging is defined as “…an increased chance of death with age, and decreased reproductive fitness with age.” One well-studied example of SAI is the removal of damaged macromolecules, a failure in which can lead to cell death. Another well-studied example of SAI is the regulated stability of stress-response factors such as SKN-1/Nrf2, which facilitates a rapid response to stress and subsequent cell survival, see Turner et al. SAI also promotes the maintenance of genetic diversity. For example, the instability of the male mitochondria relative to the nucleus in the male germline and fertilized zygote produces uniparental mitochondrial transmission. Because natural selection can only act to optimize mitochondrial gene function in the female, this is predicted to produce male-harming mitochondrial alleles, sometimes referred to as “mother’s curse” (Frank and Hurst, 1996; Gemmell et al., 2004). It is hypothesized that this will lead to selection for compensatory nuclear alleles in males (Rand et al., 2004), and that these nuclear alleles may in turn have negative effects in females (Tower, 2006). This segregating genetic diversity is proposed to include negative alleles that contribute to aging in both males and females, as well as providing a substrate for further evolution. Fisher’s principle is proposed to create a limit to the extent that male-harming or female-harming alleles might accumulate in the genome. If harming alleles accumulate to point that they reduce the viability and abundance of one sex, that sex will now have access to a greater relative abundance of possible mates. This increases the effective reproductive fitness of the limiting sex, leading to selection for production of more individuals of that sex.

Edmands reviews the experimental evidence for and against mother’s curse in detail, and discusses the sexually dimorphic and energy-intensive traits most likely to be affected. The support for mothers curse was found to be limited to a few taxonomic groups, with the strongest support coming from studies of *Drosophila*. However, other studies of *Drosophila* and other taxa failed to find support. Several factors were suggested to explain the mixed results, including the likely selection for compensatory alleles in males, as discussed above.

Sprason et al. review the role of mitochondria in aging, and in particular the role of mitochondrial DNA (mtDNA) deletions. They begin with an operational definition of aging as “…the lifelong continuous loss of physiological homeostasis resulting in a continually increasing probability of pathology and death”. mtDNA deletions increase with age in several human tissues, and mtDNA deletions are enriched in the brains of patients with Alzheimer’s disease and Parkinson’s disease, however, distinguishing between correlation and causation has proven challenging. Key studies are described in which mice were engineered with increased mtDNA mutations, and premature aging phenotypes were found to correlate with increase mtDNA deletions, but not mtDNA point mutations. By contrast, subsequent studies in various model systems produced mixed results regarding the causative effect of mtDNA deletions in aging. Possible reasons for the mixed results are suggested to include tissue-specific effects, and the limitations of current quantification methods.

Osiewacz reviews studies of the model organism *Podospora anserina* that provide insight into the role of mitochondria and biomembranes in aging. Here, aging is operationally defined as “…a complex process leading to functional degeneration and, ultimately, the death of the system.” During *P. anserina* aging, the first intron of the mtDNA gene CoxI is liberated and becomes amplified. The amplified intron sequences then act a mtDNA mutagen, leading to large mtDNA deletions. Remarkably, *P. anserina* mutants that lack the CoxI gene are viable, and do not undergo this mtDNA rearrangement. These mutants carry out an alternative form of respiration, and appear to be immortal. This alternative respiration pathway bypasses steps that normally generate abundant superoxide. Aging in *P. anserina* is also associated with pronounced alterations in the architecture of the inner mitochondrial membrane (IMM). Strikingly, mutation of certain factors that modulate membrane structure were found to increase life span. Moreover, culture of *P. anserina* with oleic acid as the carbon source altered membrane physiology and increased life span. These results causally implicate mtDNA deletions, mitochondrial superoxide, and mitochondrial membrane dynamics in *P. anserina* aging, with broader implications for our understanding of aging in humans and other organisms.

Turner et al. review the regulation and function of the *C. elegans* SKN-1 stress-response transcription factor, and the structurally and functionally related mammalian Nrf2 stress-response transcription factor. SKN-1/Nrf2 integrates a variety of internal and external stress signals, and regulates several stress responses. These responses include upregulation of detoxification genes in response to xenobiotic stress, upregulation of proteasome subunit genes in response to proteasome inhibition, and upregulation of mitophagy genes in response to disrupted mitophagy and oxidative stress. As a consequence, SKN-1 mutant animals are sensitive to acute oxidative stress and are short-lived. SKN-1 is activated by dietary restriction (DR), and normal SKN-1 function is required for life span extension by DR, as well as for life span extension caused by inhibited insulin-like signaling. SKN-1 mutations alter lipid metabolism and lipid tissue localization, and implicate SKN-1 in mediating trade-offs between reproduction and somatic maintenance.

Austad et al. review the effects of DR and amino acid restriction. First characterized in rodents, DR has since been found to increase life span across a variety of species, including *C. elegans* and *Drosophila*. DR is generally found to increase animal health and function at late ages, however, there are some side-effects observed in rodents including loss of muscle and bone mass and increased sensitivity to infections. The authors also review studies that limit individual amino acids such as methionine, tryptophan or isoleucine, as well studies that limited groups of amino acids such as branched-chain amino acids. These interventions generally recapitulated the benefits of DR, although certain benefits were dependent upon animal sex or genotype. Based upon these positive results from model systems, the authors suggest now is the time to test amino acid restriction diets in humans, to determine what might be the long-term health benefits or consequences.

da Silva et al. review the role of the gonad in *C. elegans* aging, and in particular, the negative effect of the germ-line. Ablation of the germ-line cells using a laser or genetic mutations increases worm life span and stress resistance, with greater effects observed in hermaphrodites relative to males. Intriguingly, ablation of the entire gonad does not extend life span, indicating a role for the somatic gonad. Both the cholesterol-derived hormone dafachronic acid and intestinal lipid metabolism remodeling are required for life span extension. The lipid remodeling involves activation of SKN-1 and changes to the membranes of lysosomes. Germ-line ablation also alters *Drosophila* lipid metabolism and increases life span in *Drosophila* (Flatt et al., 2008; Landis et al., 2023; Rodrigues et al., 2024).

Brandhorst and Longo state that “Aging is generally considered a time-related, non-adaptive, and deteriorating process resulting from the decline in the force of natural selection.” They review several theories of aging, including adaptive theories (sometimes referred to as “programmed aging”) as well as non-adaptive theories including antagonistic pleiotropy. They propose the term “juventology” to refer to multiple longevity programs that can be induced by DR, and that are characterized by cellular protection, optimized function and regenerative processes.

The perspective by Finch discusses the effects of air pollution on aging and the risk of dementia. Air pollution results in earlier onset of cardiovascular disease and neurodegenerative disease, and increases mortality, consistent with the conclusion that air pollution accelerates aging. These effects are exacerbated by a lower socioeconomic strata of education and wealth. Gestational environmental factors include maternal diet, and maternal exposure to air pollution and cigarette smoke. In rodent models, adult exposure to air pollution caused neuronal inflammation, and activated the expression of Nrf2-regulated detoxification genes. Gestational exposure of rodents to air pollution increased body fat, impaired glucose clearance, and decreased neurogenesis in the hippocampus. The results support the implementation of interventions in human aging and dementia based upon reducing life-long air pollution exposure.

The hypothesis and theory article by Zane et al. reviews loss of intestinal barrier integrity during aging in *Drosophila* and other species. Loss of intestinal barrier integrity can be detected by feeding animals a non-absorbable blue dye. In normal individuals the dye remains in the gut lumen, but upon loss of intestinal barrier integrity the dye moves into the circulatory system, causing the whole animal to appear blue (the “SMURF” phenotype). *Drosophila* exhibiting SMURF die within ∼48 h. Zane et al. state that “Our experiments show that every individual turns Smurf before death … ”. By contrast, three groups have reported that many *Drosophila* die without exhibiting SMURF, indicating a dependance on genotype and/or culture conditions, see Tower. Zane et al. use mathematical modeling to explore the idea of aging as a two-phase process demarked by the SMURF phenotype. In contrast to prevailing non-adaptive models, they conclude that aging is adaptive, and is “…something that has been and is directly selected, rather than a mere by-product of other processes under selection.”

The perspective on markers and mechanisms of death in *Drosophila* (Tower) discusses phenotypes that occur with several days or several hours prior to death. These include cessation of egg laying, decreased locomotor activity, increased endogenous green fluorescence, and a spike in expression of circadian rhythm genes associated with a loss of rhythmic movement. The expression of innate immune response genes and the mitochondrial unfolded protein response gene hsp22 increases progressively during aging, in the absence of any SMURF phenotype. The expression level of fluorescent transgenic reporters for these genes is partially predictive of remaining life span, even when assayed at early or mid-life timepoints. Many flies exhibit bouts of erratic behavior in the hours prior to death, often leading to falls to a supine (on the back) position. The aged fly often has difficulty righting itself from the supine position, and a fly in supine position cannot access food or water, leading to dehydration/starvation, and accelerating death. The perspective concludes that falls and loss of intestinal barrier integrity represent distinct causes of death in *Drosophila*, and that both are consistent with mitochondrial dysfunction as a possible common mechanism.

## Future perspectives

The articles emphasize the role of mitochondrial maintenance failure in aging, including the role of mtDNA deletions and mitochondrial membrane disruptions. Sexual differentiation, including germ-line cells and uniparental mitochondrial transmission, are implicated as causal factors. Lipid metabolism, as regulated by diet and SKN-1/Nrf2, emerges as one likely mechanism for trade-offs between reproduction and somatic maintenance. Environmental factors including air pollution and diet provide promising targets for interventions in human aging.
